# Genomics of Emerging Infectious Disease: A PLoS Collection

**DOI:** 10.1371/journal.pbio.1000224

**Published:** 2009-10-26

**Authors:** Jonathan A. Eisen, Catriona J. MacCallum

**Affiliations:** 1 University of California Davis, Davis, California, United States of America; 2 Public Library of Science, Cambridge, United Kingdom

Today, the Public Library of Science publishes a collection of essays, perspectives, and reviews about how genomics, with all its associated tools and techniques, can provide insights into our understanding of emerging infectious disease (http://ploscollections.org/emerginginfectiousdisease/) [1]–[13]. This collection, focused on human disease, is particularly timely as pandemic H1N1 2009 influenza (commonly referred to as swine flu) spreads around the globe, and government officials, the public, journalists, bloggers, and tweeters strive to find out more. People want to know if this flu poses more of a threat than other seasonal flu strains, how fast it's spreading (and where), and what can be done to contain it. As this collection illustrates, the increasing speed at which complete genome sequences and other genome-scale data can be generated for individual isolates and strains of a pathogen provides tremendous opportunities to identify the molecular changes in these disease agents that will enable us to track their spread and evolution through time (e.g., [3],[7],[8]) and generate the vaccines and drugs necessary to combat them (e.g., [5]–[7]). The collection also shines a spotlight on specific pathogens, some familiar and widespread, such as the influenza A virus (e.g., [9]); some “reemerging,” such as the *Mycobacterium tuberculosis* complex that causes tuberculosis [10]; and some identified only recently, as with the bacterium *Helicobacter pylori* (which causes peptic ulcers and gastric cancer [11]).

There is no simple definition of an emerging disease, but it can be loosely described as a disease that is novel in some way—for example, one that displays a change in geographic location, genetics, or function. Emerging infectious diseases are caused by a wide range of organisms, but they are perhaps best typified by zoonotic viral diseases that cross from animal to human hosts and can have a devastating impact on human health, causing a high disease burden and mortality [8]. These zoonotic diseases include monkeypox, Hendra virus, Nipah virus, and severe acute respiratory syndrome coronavirus (SARS-CoV), in addition to influenza A and the lentiviruses that cause AIDS. The apparently increased transmission of pathogens from animals to humans over the recent decades has been attributed to the unintended consequences of globalization as well as environmental factors and changes in agricultural practices [8]. Generally, the burden of these diseases is most strongly felt by those in developing countries. Brindley et al. [12] point to the debilitating effects of the most common human infectious agent in such areas—helminths (parasitic worms)—and the role that genomics plays in advancing our understanding of molecular and medical helminthology. Compounding the problem of emerging infectious diseases in developing countries is the reality that researchers in developing countries have often been unable to participate fully in genomics research, because of their technological isolation and limited resources. As Harris et al. emphasize [13], “collaborations—starting with capacity building in genomics research—need to be fostered so that countries that are currently excluded from the genomics revolution find an entry point for participation.”

This collection is a collaborative effort that combines financial support from Google.org (which has also sponsored research on emerging infectious disease through its Predict and Prevent initiative [14]) with PLoS's editorial independence and rigor. Gupta et al. [1] provide Google.org's perspective and vision for how systematic application of genomics, proteomics, and bioinformatics to infectious diseases could predict and prevent the next pandemic. To realize this vision, they urge the community to unite under an “Infectious Disease Genomics Project,” analogous to the Human Genome Project. This is, as the authors admit, a potentially “grandiose” and difficult proposition. Some researchers might justifiably argue that much is already being achieved—as demonstrated by this collection—and that the vision is naïve. However, as every article in the collection also points out, tremendous challenges remain if the potential of genomics in this field is to be realized.

One problem is that, despite the fact that sequencing is now the method of choice for characterizing new disease agents, and new substantially faster and cheaper sequencing methods are continually being produced, we still lack the range of computational tools necessary to analyze these sequences in sufficient detail [4]. It is possible to sequence the entire assemblage of viruses in a particular tissue type or host species [3] and to obtain complete or nearly complete genome sequences for large samples of bacteria [7]. Yet we remain in the early, albeit essential, stages of pathogen discovery (Box 1). These sequences can be interpreted fully only when integrated with relevant environmental, epidemiological, and clinical data (e.g., [3],[4],[8]). And, despite the increased sequencing, really comprehensive genome data are still only available for a few key pathogens, which further limits our understanding. For example, a full quantitative understanding of the processes that shape the epidemiology and evolution—the phylodynamics—of RNA viruses is currently possible only for HIV and influenza A virus [3].

**Box 1. A Field Guide to Microbes?**

When an American robin (*Turdus migratorius*) showed up in London a few years ago, birders were rapidly all atwitter and many came flocking to town [22]. Why had this one bird created such a stir? For one main reason—it was *out of place*. This species is normally found in North America and only very rarely shows up on the other side of the “pond.” Amazingly, this rapid, collective response is not that unusual in the world of birding. When a bird is out of place, people notice quickly.

This story of the errant robin gets to the heart of the subject of this collection because being *out of place* in a metaphorical way is what defines an emerging infectious disease. Sometimes we have never seen anything quite like the organism or the disease before (e.g., SARS, *Legionella*). Or perhaps, as with many opportunistic pathogens, we have seen the organism before but it was not previously known to cause disease. In other cases, such as with as pandemic H1N1 2009 or *E. coli* O157:H7, we have seen the organism cause disease before but a new form is causing far more trouble. And of course organisms can be literally out of place, by showing up in a location not expected (e.g., consider the anthrax letters [2]).

Historically, despite the metaphorical similarities with the robin case, the response to emerging infectious disease is almost always much slower. Clearly, there are many reasons for these differences, which we believe are instructive to consider. At least four factors are required for birders' rapid responses to the arrival of a vagrant bird: (1) knowledge of the natural “fauna” in a particular place, (2) recognition that a specific bird may be out of place, (3) positive identification of the possibly out-of-place bird, and (4) examination of the “normal” place for relatives of the identified bird.

How are these requirements achieved? Mostly through the existence of high-quality field guides that allow one to place an organism such as a bird into the context of what is known about its relatives. This placement in turn is possible because of two key components of field guides. First, such guides contain information about the biological diversity of a group of organisms. This usually includes features such as a taxonomically organized list of species with details for each species on biogeography (distribution patterns across space and time, niche preferences, relative abundance), biological properties (e.g., behavior, size, shape, etc.), and genetic variation within the species (e.g., presence of subspecies). Second, a good field guide provides information on how to identify particular types (e.g., species) of those organisms. With such information, and with a network of interested observers, an out-of-place bird can be detected with relative ease.

In much the same way, a field guide to microbes would be valuable in the study of emerging infectious diseases. The articles in this collection describe what can be considered the beginnings of species-specific field guides for the microbial agents of emerging diseases. If we want to truly gain the benefits that can come from good field guides it will be necessary to expand current efforts to include more organisms, more systematic biogeographical sampling, and more epidemiological and clinical data. But the current efforts are a great start.[Fig pbio-1000224-g001]

**Figure pbio-1000224-g001:**
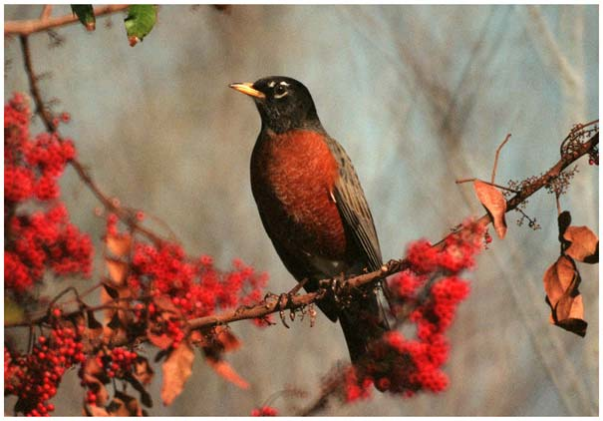
The American Robin (*Turdus migratorius*). (Photo Credit: NASA).

In this collection, you will find not only the views of leading researchers from several different disciplines, and a provocative vision from a funding agency, but also the contributions of six different PLoS journals (*PLoS Biology*, *PLoS Medicine*, *PLoS Computational Biology*, *PLoS Genetics*, *PLoS Neglected Tropical Diseases*, and *PLoS Pathogens*). The PLoS open-access model of publishing makes possible such a large multidisciplinary cross-journal collection, in which all articles are simultaneously available online for unrestricted reuse, regardless of venue (see also the podcast that accompanies the collection; http://ploscollections.org/podcast/emerginginfectiousdisease.mp3).

Our aim is that this collection will add to other “open science” activities that have helped provide insights into infectious disease more quickly than would have been thought feasible only a few years ago. This accelerated availability of research findings is exemplified by the recent response to the flu pandemic. Consider, for example, data access. Traditionally, scientists have released data after publishing a study. Fortunately, in part due to experience from genome sequencing projects, prepublication flu sequence data have been released in a relatively unrestricted manner to the community [15]. This has in turn enabled anyone—not just those who collected the data—to carry out analyses while the epidemic is occurring (when in principle there is still time to save lives) rather than being forced to provide a posthumous account of the spread of infection. Such a response highlights both the importance of early data access and the removal of restrictions in the use of data (e.g., in many past cases data might be released but use of the data in presentations and publications would be limited).

The value of open access to sequence data is helping to put pressure both on private organizations to release their sequence data [16],[17] and on all agencies to release other information (e.g., metadata about strains) more rapidly. This pressure is not being brought to bear only on flu data—in this collection Van Voorhis et al. [5] call on pharmaceutical companies to deposit the structural coordinates of drug targets from all globally important infectious disease organisms in public databases.

Of course, data about any infectious disease are not very useful unless placed in the scientific context of past studies (i.e., publications) specifically about the disease or about methods to analyze such data. It is also important to have access to information about other diseases and other organisms that might impact its spread or evolution. Perhaps the most intriguing aspect of open science in response to flu has been the move toward pre-journal publication release of findings. Many flu researchers took the available data, analyzed it, and posted results on blogs [18],[19], wikis [20], and other sites. Although some view this “non peer-reviewed” release as unseemly, it is clear that it has helped accelerate the science in the study of pandemic H1N1 2009 and led to some important journal papers [17]. Indeed, such advances helped provide one of the stimuli for PLoS's most recent initiative, PLoS Currents: Influenza, a Google “Knol,” for the rapid communication of research results and ideas about flu vetted by expert moderators [21].

This is not to say there are no possible risks or drawbacks from more openness. For example, some governments may avoid releasing data because of fears about discrimination (as was seen in many aspects of the flu in Mexico). Others worry that complete openness might foster the spread of misinformation. However, as Fricke et al. argue in their article on the relationship between genomics and biopreparedness [2], open source genomic resources are actually of immense benefit to those in charge of our public health and biosecurity.

It is clear that “for all stages of combating emerging infections, from the early identification of the pathogen to the development and design of vaccines, application of sophisticated genomics tools is fundamental to success” [8]. It is equally clear that open science and open access to publications and data will be key to that success. Whatever one's position has been on the various open science initiatives, there is no doubt that the “esoteric” label on some open science initiatives has largely been eliminated by the emergence of H1N1 flu epidemic.

The faster, cheaper, and more openly we can distribute the discoveries of science, the better for scientific progress and public health. As this collection emphasizes, managing the threat of novel, re-emerging, and longstanding infectious diseases is challenging enough even without barriers to scientific research. We encourage you to make the most of this collection by sharing, rating, and annotating the articles using our online commenting tools. Better yet, join the discussion by providing your own vision to prevent the emergence and spread of the next rogue pathogen.
